# Understanding for whom, under what conditions, and how group-based physical activity interventions are successful: a realist review

**DOI:** 10.1186/s12889-015-2270-8

**Published:** 2015-09-24

**Authors:** Samantha M. Harden, Desmond McEwan, Benjamin D. Sylvester, Megan Kaulius, Geralyn Ruissen, Shauna M. Burke, Paul A. Estabrooks, Mark R. Beauchamp

**Affiliations:** Human Nutrition, Foods and Exercise, Virginia Tech, 1981 Kraft Dr., Room 1009, Blacksburg, VA 24060 USA; University of British Columbia, School of Kinesiology, 6081 University Blvd, Vancouver, BC V6T 1Z1 USA; School of Health Studies, Western University, 1151 Richmond St, Room 216, HSB, London, ON N6A 3 K7 USA; Human Nutrition, Foods and Exercise, Virginia Tech, Family and Community Medicine, Carilion Clinic, 1 Riverside Circle, Roanoke, VA 24016 USA

**Keywords:** Group-based, Physical activity, Realist review, Interventions

## Abstract

**Background:**

Participation in group-based physical activity (GBPA) interventions has been found to result in higher levels of exercise adherence and program compliance. However, previous reviews of GBPA programs have provided limited insight regarding ‘for whom’, ‘under what conditions’, and ‘how’ these interventions increase physical activity behavior.

**Methods:**

A realist review was conducted by following the seven recommended iterative and overlapping steps (J Health Serv Res Policy 10S1:21-34, 2005). The review was limited to group dynamics-based interventions for adults (>17 years of age). The search was conducted in PubMed, PsychInfo, and Web of Science search engines associated with the Science Citation Index Expanded, Social Sciences Citation Index, Arts & Humanities Citation Index, and MEDLINE.

**Results:**

Using a realist review approach, data from 52 studies were synthesized. Of those, 92 % (*n =* 48) reported significant increases in participant physical activity. The synthesis resulted in three main observations and recommendations.

**Discussion:**

GBPA interventions have worked for a variety of populations, including those who are hard to reach; however, more research is needed on moderating factors to determine for whom different GBPA programs may be effective. Second, previous interventions have varied in the duration, frequency, and number of group-based strategies used, and comparative effectiveness research may be necessary to isolate the mechanisms of effect. Third, these interventions have been conducted in a diverse range of settings, using a variety of research designs and analytical approaches. Less information is known about the costs or sustainability of these programs in their intended settings.

**Conclusion:**

The results of this realist review have important implications for practice, refining trial designs, and replication across diverse populations and settings.

**Electronic supplementary material:**

The online version of this article (doi:10.1186/s12889-015-2270-8) contains supplementary material, which is available to authorized users.

## Background

The prevalence of physical inactivity is a worldwide problem of growing proportions [12, 51]. In order to increase physical activity participation across the lifespan, a wide array of intervention approaches have been employed. One such approach is the use of group dynamics to improve physical activity among individuals in groups of various sizes ranging from small teams to communities.

Increasing physical activity using a group dynamics approach has been effective with various populations such as women within the prenatal period [14, 15, 32], minority populations [38, 39], older adults [9, 24, 49] as well as individuals with a chronic condition [35]. However, a limited number of moderation analyses have been conducted by researchers to understand the boundary conditions for whom these interventions may be more or less successful [25]. That is, group-based physical activity programs may be more effective for certain populations, and data around boundary conditions could help practitioners in determining both the appropriateness of, and recommended strategies for, a group-based approach.

From a setting-level, previous group-based interventions targeting physical activity have been successfully delivered in worksites [57], faith-based organizations [38], and in the community at large [22]. Environmental factors such as accessibility, opportunities, and aesthetic attributes of the environment, as well as policies within said environment, significantly influence physical activity participation [7, 36]. However, less is known about the conditions under which these programs were effective, including information related to environmental factors (e.g., location) as well as the individuals responsible for the optimal delivery of the intervention (e.g., coordinator, champion, leader; [23]). Across the general physical activity domain, effective leader characteristics include resource deployment, provision of feedback, and demonstrating credible knowledge and skills related to physical activity. From a translational perspective, it is notable that the level of physical activity participation of care providers [41] and community health educators [27] has been related to their advocacy of the health behavior in others. As such, the personal characteristics of those delivering an intervention may play a role in program adoption and treatment fidelity. However, the studies that include characteristics of those delivering the program remains low, making it difficult to discern who to recommend (and what characteristics they might possess) for the delivery of an intervention and how to best influence their decision to adopt a program.

There is also a lack of information about the underlying mechanisms by which group-based physical activity interventions might be successful. Behavioral constructs such as the need to belong [3], social support [29], friendly competition [34] and/or motivational effects [37], and group cohesion [10] have been cited as influencing physical activity behavior change. However, in a recent systematic review of group-based physical activity interventions for adults, it was reported that only one study conducted a mediation analysis (i.e., perceptions of cohesion mediated the relationship between GBPA and resultant physical activity levels; [25]). Therefore there are limited data on the extent to which particular strategies mediate the effects of group-based physical activity interventions.

A comprehensive analysis regarding ‘for whom’, ‘under what conditions’, and ‘how’ group-based physical activity interventions are effective has yet to be conducted. Evidence on the environmental (e.g., location), situational (e.g., context), and implementation (e.g., delivery agent) factors related to the delivery of group-based interventions is largely missing in the literature and is needed to improve and inform evidence-based physical activity promotion [8]. Group-based strategies used to promote physical activity that account for both systemic and individual factors have the potential to be more impactful. Indeed accounting for contextual and individual factors are more likely to be adopted, effective, and sustained compared to interventions that do not account for these factors [31]. In fact, findings from Estabrooks et al. [25] indicate that there is a paucity of information in the physical activity promotion literature around the best strategies to employ within particular groups.

One methodological approach to answer these more complex questions is to conduct a realist review. Realism is based in philosophy and has been applied to various scientific fields such as economics and sociology. Although this approach has been less prevalent in synthesizing evidence in health psychology [45], it has the potential to provide valuable information for this field. A realist review can be used to answer how, where, and why a disparate range of interventions work (or not).

The realist review provides a means to deal with intervention heterogeneity (e.g., study design, outcome measures) and make inferences about context and effectiveness [45]. As there is very little information on the underlying mechanisms of behavior change (i.e., mediation analyses) within group-based physical activity interventions [25], a realist review has the potential to inform the development of a conceptual framework that describes the context (both populations and settings) as well as the approach under which group-based physical activity programs have been effective.

A realist approach could be used to identify gaps in our understanding of group-based physical activity interventions and provide suggestions for future research. Numerous systematic and meta-analytic reviews have concluded that group-based physical activity programs have a large effect on increasing physical activity [18] and are successful at increasing perceptions of cohesion, which ultimately lead to greater participant adherence and compliance [9, 21]. In spite of these advances there remains a lack of understanding with regard to ‘for whom,’ ‘under what conditions,’ and ‘how’ these interventions are successful. While the *a priori* aim of measuring effectiveness of interventions is warranted, such an approach is typically insufficient for knowledge translation within complex systems [50]. In fact, reflective information about for whom, under what conditions, and how group-based physical activity interventions work has the potential to substantively contribute to the degree to which effective interventions may be translated, and sustained, in practice. Using a realist approach, the purpose of the current study was to identify for whom, under what conditions, and how group-based physical activity interventions are effective.

## Methods and results

The review was conducted by following the seven iterative and overlapping steps recommended by [45]. The first two steps defined and refined the scope of the review and included a search for relevant evidence, respectively. Subsequently, in the third step, the resultant studies were appraised to determine the degree to which they informed the scope of the review. In the fourth step, data related to the aims of the study were extracted from each article. In the fifth step, data were synthesized to develop evidence-based suggestions for group dynamics-based physical activity interventions. The final two steps in a realist review are to disseminate findings and provide formative data for refining large-scale programs in appropriate contexts (i.e., the intent of this manuscript).

### Step 1: Define the scope of the review

The scope of this review was to answer the following questions: (1) For whom (e.g., participant age, sex, activity level) are group dynamics-based physical activity interventions effective?; (2) Under what conditions (e.g., research design, program location, measures) are these interventions successful?; and (3) How (e.g., theoretical foundation, group processes, group structure) are these interventions successful? For the purpose of this review, an intervention was deemed ‘effective’ if there was a significant increase in physical activity, exercise, fitness, and/or program adherence (hereafter referred to as physical activity behavior). Adherence to the physical activity program was a measure of physical activity behavior. That is, participants were physically active while participating in the group sessions.

**Fig. 1:**
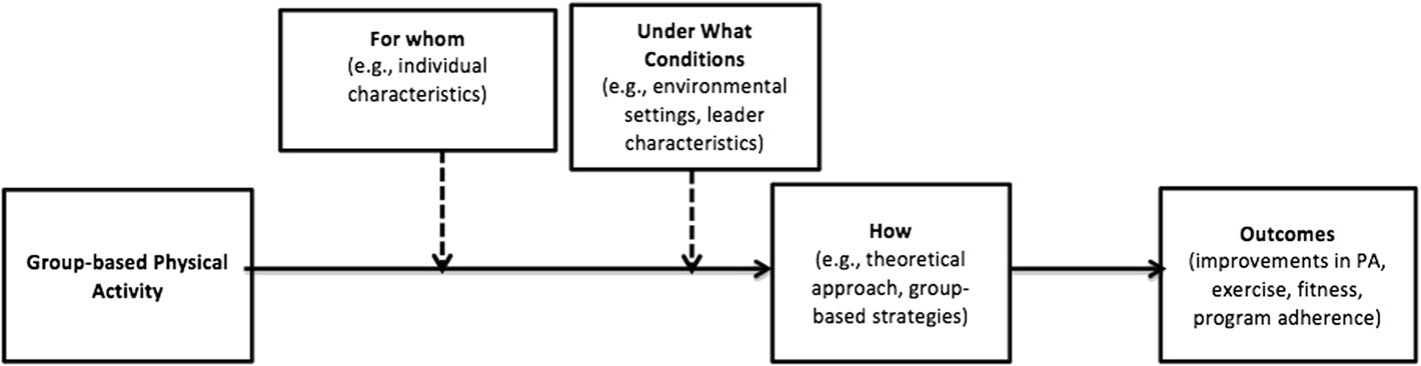
Contextual framework for group-based physical activity promotion interventions

The conceptual basis that framed the inter-relationships between these three broad research questions is displayed in Fig. 1. The review was limited to adult populations (i.e., target audience of those who were >17 years of age). The scope of this review was also limited to group dynamics-based interventions, rather than any physical activity intervention delivered to an aggregate of people. This decision was guided by the conceptualization of ‘true groups’ in exercise settings as outlined by Burke and colleagues [9]. Specifically, Burke et al. [9] posited that exercise classes in which evidence-based group-dynamics principles are employed to enhance cohesiveness can be considered true groups [54]. A recent definition of a ‘true group’ indicated the following criteria must be met: (a) an individual is changed (e.g., behaviors, experiences, self-identification) by joining the group; (b) interactions with group members both influences oneself and the other members; (c) the group is attracted to a common goal; and (d) members within the group identify as a group [26]. A table representing eligibility criteria is available as a Additional file 1.

### Step 2: Search for relevant evidence

The authors identified key strategies and principles embedded within pre-existing group-based physical activity models including Carron and Spink’s multidimensional conceptual model of team building [11], Brawley and colleagues’ group-mediated cognitive behavioral (GMCB) approach [5], and a model for teamwork and team effectiveness in sport groups [43]. The group dynamics terms that were used in the database search can be found in Table 1. Each of these terms was searched for in conjunction with ‘physical activity’, ‘exercise’, ‘adherence’, and ‘fitness’. These search terms were chosen to allow for a feasible scope of identifying true groups, rather than any physical activity intervention delivered to a group of people.

**Table 1:** Theory-based search terms

Model	Constructs	Search terms	
Team Building Model (Carron & Spink, 1992)	▪Group cohesion	▪“Group cohesion”	▪Competit*
	▪Group structure	▪“Group structure”	▪Group problem solving
	▪Group roles	▪“Group roles”	▪Collective efficacy
	▪Group norms	▪“Group norms”	▪Group communication
	▪Group environment	▪“Group environment”	▪“Communication” AND “group”
	▪Distinctiveness/team identity	▪Distinct*	
	▪Group size	▪Team ident*	
	▪Leadership	▪“Group size”	
	▪Group processes	▪Leadership	
	▪Group goals	▪Group process*	
	▪Cooperation	▪“Group goals”	
	▪Competition	▪“Cooperation”	
	▪Group problem solving		
	▪Collective efficacy		
Group-mediated cognitive-behavior intervention (Brawley, Rejeski, & Lutes, 2000)	▪Distinctiveness	▪Distinct*	
	▪Discussion of physical activity behaviors	▪Discussion of behaviors	
	▪Self-regulatory skills	▪Self-regulat*	
	▪Outcome expectations	▪Tapering of group*	
	▪Tapering of groupness	▪Group terminat*	
	▪Social pressure	▪“Social pressure”	
	▪Support for group goals	▪“Support” AND “group goals”	
		▪Groupness	
Teamwork Model (McEwan & Beauchamp, 2014)	▪Psychological support	▪“Psychological support”	▪“Intrateam coaching”
	▪Coordination	▪“Coordination”	▪“Coach*” AND “group”
	▪Performance monitoring	▪Performance monitor*	▪Innovation
	▪System monitoring	▪System monitor*	▪Integrative conflict manag*
	▪Backing up	▪“Backing up”	▪“Emotional support”
	▪Intrateam coaching	▪“Task support”	
	▪Innovation		
	▪Integrative “conflict management”		
	▪Emotional support		

*An asterisk associated with a search term indicates proximity searches. For example "Distinct*" would capture both distinctiveness and distinction

Our search strategy included the use of the following search engines and databases: PubMed, PsychInfo, and Web of Science search engines associated with the Science Citation Index Expanded, Social Sciences Citation Index, Arts & Humanities Citation Index, and MEDLINE. The search was completed in August 2013 and data analysis was completed in November 2014.

**Fig. 2:**
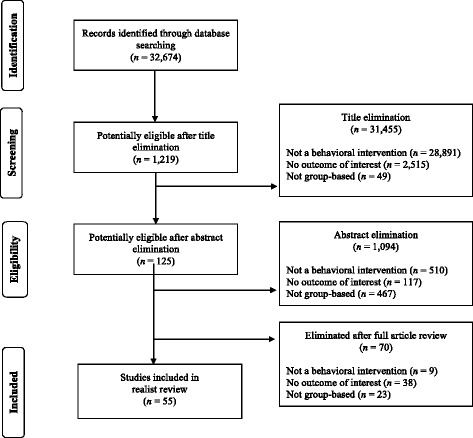
Results of literature search

As seen in Fig. 2, the search criteria resulted in 32,674 unique and potentially eligible articles. Title elimination was conducted by the first author, and confirmed by the second and third authors. Pairs of the first five authors independently reviewed abstracts and eliminated 1,094 articles for reasons displayed in Fig. 2. Subsequently, 70 articles were excluded after two of the first five authors independently reviewed the full manuscript (the primary reason for exclusion was the absence of a behavioral intervention). Thus, 56 articles were included in the final realist synthesis. The PRISMA diagram is demonstrated in Fig. 2, citations for all articles available in Additional file 2. One study was represented three times (i.e., across three articles) and two studies were represented twice (i.e., in two articles); therefore, there were a total of 52 unique group-based physical activity interventions evaluated in this review.

### Step 3: Study quality appraisal

Integral to the data synthesis step, a quality appraisal was conducted of the eligible articles. Standard quality appraisal checklists have been identified as insufficient for realist reviews [45]. That is, a hierarchical approach (with a randomized controlled trial taking precedence over all other methodologies, from non-randomized designs to case studies) often lacks the ability to consider complex systems [45]. In fact, ‘gold-standard’ quality assessments are counterintuitive to the realist approach that integrates internal and external validity factors. Drawing from Pawson et al. [45], the quality appraisal used in the current study was comprised of two dichotomous items (1 = yes; 0 = no) for relevance and rigor. The purpose of these two items was to determine if the study fit within the scope of this review (relevance) and if the conclusions drawn by the researchers aligned with their research design (rigor). Only those studies that demonstrated both relevance and rigor (i.e., scored 2) were included in this review.

### Step 4: Data extraction for realist review

The lead author and one randomly assigned co-author completed the data extraction process for the first 33 articles. To promote consistency among coders, authors were provided with a coding companion guide with operational definitions of each variable. Indicators were adapted from coding sheets developed for previous reviews of group-based physical activity interventions (cf. [9, 25, 33]). The data extraction tool of the latter two studies included factors related to the Pragmatic-Explanatory Continuum Index Summary (PRECIS; [55]) and the Reach, Effectiveness, Adoption, Implementation, and Maintenance (RE-AIM) framework [31, 48] in order to encompass individual and system-level factors as described below. The coding authors met to resolve discrepancies by referring back to the article. As inter-rater reliability was over 90 % for the first 33 articles, two randomly assigned authors coded the remaining 19 articles. Inter-rater reliability between coders remained high (>90 %) with the co-authors again meeting to resolve any outstanding discrepancies. The authors met to collectively establish the indicators based on operational definitions of each theme.

Generally speaking, ‘for whom’ variables included descriptors of the individuals or groups to whom group-based physical activity interventions have been delivered. We captured 10 indicators: target population descriptors, racial composition, inclusion criteria, exclusion criteria, sample size, participation rate, sex, age, fitness level (pre-intervention), and health status (see Table 2).

**Table 2:** For whom GBPA interventions are effective

	Demographics	Eligibility criteria	Sample	Fitness level	Health status
Positive effect group-versus-individuals/aggregate (*n* =39)	*M* Age (SD) 54.01 (±14.91)	Inclusion Criteria 2.69 (±1.96)	Reported (*n*=13)	Sedentary (*n*=8)	Healthy (*n* =12)
				Insufficiently active (*n* =3)	At risk (*n* =2)
	Sex	Exclusion Criteria 1.17 (±1.65)	Average Participation Rate: 15 %	Active (*n* =3)	With chronic condition (*n* =4)
	Male (*n*=2)		Sample Size *M* (SD) 1135.13 (±3207.26)	Mix (*n* =8)	Both health and at risk (*n* =2)
	Female (*n* =11)				
	Mixed (*n* =26)			Not reported (*n* =17)	Both healthy and have chronic condition (*n* =0)
	Race				Not reported (*n* =19)
	Mixed Racial Composition (*n* =35)				
	Primarily Minority (*n*=4)				
Group-versus-group interventions (*n*=9)	*M* Age (SD) 53.57 (±13.78)	Inclusion Criteria 1.44 (±1.13)	Reported (*n*=6)	Sedentary (*n* =2)	Healthy (*n* =0)
	Sex	Exclusion Criteria 3.11 (±1.83)	Average Participation Rate: 28 %	Insufficiently active (*n* =2)	At risk (*n* =3)
	Male (*n* =1)				
	Female (*n* =4)		Sample Size *M* (SD) 165.89 (±111.41)	Active (*n* =1)	With chronic condition (*n* =3)
	Mixed (*n* =4)				
	Race			Mix (*n* =0)	Both health and at risk (*n* =2)
	Mixed Racial Composition (*n* =5)			Not reported (*n* =4)	Both healthy and have chronic condition (*n* =1)
	Primarily Minority (*n* =4)				Not reported (*n* =0)
No increase in physical activity (*n*=4)	*M* Age (SD) 57.81 (±5.99)	Inclusion Criteria 1.00 (±1.15)	Reported (*n*=2)	Sedentary (*n* =1)	At risk and has chronic condition (*n* =2)
				Insufficiently active (*n* =1)	Not reported (*n* =2)
	Sex	Exclusion Criteria 3.75 (±3.30)	Average Participation Rate: 21 %		
	Male (*n* =1)				
	Female (*n* =1)		Sample Size *M* (SD) 112.33 (±112.29)	Active (*n* =0)	
	Mixed (*n* =2)				
	Race			Mix (*n* =0)	
	Mixed Racial Composition *(n*=1)			Not reported (*n* =2)	
	Primarily Minority (*n*=3)				

‘Under what conditions’ variables included those associated with the study design and the context in which the study took place. The thirteen indicators were: research design, recruitment methods, use of physician referral, sample selection, program location, setting sample size, setting adoption rate, country in which the research was conducted, costs of implementation, type of measurement, type of analysis, moderation analysis, and post-intervention assessment time point (see Table 3).

**Table 3:** Under what conditions group-based physical activity interventions are effective

	Design	Recruitment procedures	Program setting	Measures
Positive effect group-versus-individuals/aggregate (*n* =39)	Randomized with control (*n* =13)	Recruitment Strategies	Program Location	Physical Activity Outcome
		Mass or local media (*n*=11)	Medical center (*n* =3)	Subjective (*n* =23)
	Quasi-experimental with control (*n* =8)	Word of Mouth (*n*=3)	Fitness facility (*n* =4)	Objective (*n* =6)
		Physician Referral (*n*=5)	University (*n* =3)	Mix (*n* =10)
	Quasi-experimental without control (*n* =3)	Flyers (*n*=11)	Worksite (*n* =5)	ITT
	Pre-post design (*n* =15)	Center-based (*n*=7)	General community (*n*=5)	Yes (*n* =6)
		Employer’s Worksite (*n*=5)	Faith-based location (*n* =2)	No (*n* =39)
		Faith-based (*n*=5)	Center-based (*n* =3)	Reported Costs
		Target Mailings (*n*=16)	Other (*n* =7)	Yes (*n* =5)
		Targeted Contact (*n*=8)	Not reported (*n* =7)	No (*n* =34
		Presentation/Seminar (*n*=8)	Country	Timing of Post-Program Assessment
		Other (*n*=2)	USA (*n* =33)	14.75 weeks (±31.69)
		Not Reported (*n*=2)	Canada (*n* =3)	Moderation Analyses
		Sample Selection	Middle East (*n* =2)	(*n*=4 interventions)
		Convenience Sampling (*n* =2)	Australia (*n* =1)	Gender (*n*=2)
		Random Selection (*n* =5)	Average Setting Sample Size	Age (*n*=1)
		Targeted (*n* =26)	17.76 (±31.39)	Baseline activity level (*n*=2)
		Convenience and Targeted (*n* =1)	Average Setting Adoption Rate	Self-monitoring (*n*=1)
		Random and Targeted (*n* =3)	77.25 % (±25.55 %)	Member Diversity (*n*=1)
		Not specified (*n*=2)	Not report (*n* =31)	Perceived health (*n*=1)
		Physician Referral		Distance to Group Sessions (*n*=1)
		Yes (*n*=5)		Socio-economic status (*n*=2)
		No (*n* =34)		
Group-versus-group interventions (*n* =9)	Randomized with control (*n* =3)	Recruitment Strategies	Program Location	Physical Activity Outcome
		Mass or local media (*n*=2)	Medical center (*n* =3)	Subjective (*n*=2)
		Word of Mouth (*n*=3)	Faith-based location (*n* =2)	Objective (*n*=0)
	Quasi-experimental with control (*n* =4)	Physician Referral (*n*=2)	Nursing home (*n* =1)	Mix (*n*=7)
		Flyers (*n*=3)	Cooperative extension (*n* =1)	ITT
		Center-based (*n*=1)	Not reported (*n* =2)	Yes (*n* =2)
		Employer’s Worksite (*n*=1)	Country	No (*n*=7)
	Quasi-experimental without control (*n* =1)	Faith-based (*n*=1)	USA (*n* =7)	Reported Costs
		Target Mailings (*n*=2)	Canada (*n* =2)	Yes (*n*=1)
		Targeted Contact (*n*=3)	Average Setting Sample Size	No (*n* =8)
		Other (*n*=1)	4.17 (±3.82)	Moderation Analyses
	Pre-post design (*n* =1)	Not Reported (*n*=1)	Average Setting Adoption Rate	(*n*=0)
		Sample Selection	Not report (*n* =9)	
		Convenience Sampling (*n* =2)		
		Random Selection (*n* =0)		
		Targeted (*n* =6)		
		Convenience and Targeted (*n* =1)		
		Random and Targeted (*n* =0)		
		Not specified (*n* =0)		
		Physician Referral		
		Yes (*n* =2)		
		No (*n* =7)		
No increase in physical activity (*n*=4)	Randomized with control (*n* =3)	Recruitment Strategies	Program Location	Physical Activity Outcome
		Word of Mouth (*n*=1)	Medical center (*n* =1)	Subjective (*n* =4)
		Flyers (*n*=3)	Worksite (*n* =1)	Objective (*n* =0)
	Quasi-experimental with control (*n* =1)	Center-based (*n*=1)	Faith-based location (*n*=1)	Mix (*n* =0)
		Target Contact (*n*=1)	Not reported (*n*=1)	ITT
		Sample Selection	Country	Yes (*n* =1)
		Convenience Sampling (*n* =0)	USA (*n* =3)	No (*n* =3)
		Random Selection (*n* =0)	Australia (*n* =1)	Reported Costs
	Quasi-experimental without control (*n* =0)	Targeted (*n* =3)	Average Setting Sample Size	Yes (*n* =1)
		Convenience and Targeted (*n*=1)	102.67 (±173.50)	No (*n* =3)
		Random and Targeted (*n* =0)	Average Setting Adoption Rate	Timing of Post-Program Assessment
		Not specified (*n* =0)	50.50 %	33.00 weeks (±26.20)
		Physician Referral	Not report (*n* =3)	Moderation Analyses
	Pre-post design (*n* =0)	Yes (*n* =1)		(*n*=0)
		No (*n* =3)		

Finally, ‘how’ variables included those associated with the delivery of the intervention itself (e.g., theory that guided the intervention, mode of intervention delivery). Eleven indicators were used for this theme: duration of intervention, participant fidelity to program sessions, participant fidelity to recommended behavioral strategies, contact type, contact duration, type of physical activity recommended, who delivered the intervention, modifications targeted at environmental or social structures (or both), intervention strategies employed, theoretical approach, and mediational analyses (see Table 4).

**Table 4:** How group-based physical activity interventions are effective

	Program description		Theory	Strategies
Positive effect group-versus-individuals/aggregate effect (*n* =39)	Intervention Duration	Delivery Staff	Theory	Group Structure
	16.28 weeks (±14.55 weeks)	Research assistant (*n*=2)	Team-building (*n*=2)	Status of group members (*n* =9)
	Fidelity to Classes	Health professional (*n*=4)	Social Cognitive Theory (*n* =13)	Group roles (*n* =7)
	79.41 % (±10.98)	Extension agent (*n*=3)	Group-mediated Cognitive	Group norms (*n* =14)
	Not reported (*n*=23)	Group exercise leader (*n*=5)	Behavioral Model (*n*=6)	Group Environment
	Fidelity to Behavioral Components	Community leader (*n*=4)	Social support Framework (*n*=5)	Distinctiveness/Team Identity (*n* =12)
	83.01 % (±11.05)	Onsite coordinator (*n*=3)	Goal Setting Theory (*n*=3)	Group Size (*n* =18)
	Not reported (*n*=33)	Trained peer leader (*n*=7)	Transtheoretical Model (*n*=2)	Leadership (*n* =12)
	Average Total Contact Duration	Health professional and community leaders (*n*=1)	Social Ecological Theory (*n*=2)	Proximity (*n* =17)
	65.75 hours (±84.59)	Research assistant and group exercise leader (*n* =1)	Social Determinants of Health (*n* =9)	Group Processes
	Type of physical activity prescribed		Other (*n* =4)	Group goals (*n* =12)
	Aerobic (*n*=14)	Not reported (*n* =9)	Single theory (*n* =11)	Cooperation (*n* =4)
	Strength Training (*n* =1)	Contact Type	Multiple theory (*n* =7)	Competition (*n* =10)
	Combination (*n*=3)	In-Person (*n* =21)	Atheoretical (*n* =21)	Interaction and communication (*n* =25)
	Not reported (*n* =21)	In-Person plus another mode (*n* =11)	Targeted Structural Modification	Social Support (*n*=22)
		Non-In-Person (*n* =5)	Social (*n*=29)	Individual Strategies
		Not specified (*n* =1)	Environmental (*n*=1)	Goal setting (*n*=25)
			Both (*n*=9)	Action planning (*n* =11)
				Problem solving (*n* =12)
				Self-monitoring (*n* =22)
				Feedback (*n* =3)
Group-versus-group interventions (*n* =9)	Intervention Duration	Delivery Staff	Team-building (*n* =2)	Group Structure
	30.00 weeks (±14.283 weeks)	Research assistant (*n* =3)	Social Cognitive Theory (*n* =3)	Status of group members (*n* =1)
	Average Total Contact Duration	Health professional (*n* =1)	Transtheoretical Model (*n* =1)	Group roles (*n* =3)
	17.37 hours (±23.65)	Group exercise leader (*n* =1)	Social Determinants of Health (*n* =1)	Group norms (*n*=3)
	Not reported (*n*=1)	Trained peer leader (*n* =3)		Group Environment
	Fidelity to Classes	Research assistant and Extension agent (*n* =1)	Single theory (*n* =3)	Distinctiveness/Team Identity (*n* =3)
	66.93 % (±10.98)	Contact Type	Multiple theory (*n* =3)	Group Size (*n* =3)
	Not reported (*n*=23)	In-Person (*n*=4)		
	Fidelity to Behavioral Components	In-Person plus another mode (*n*=2)	Atheoretical (*n* =3)	Leadership (*n* =5)
	Not reported (*n*=9)	Non-In-Person (*n* =0)	Targeted Structural Modification	Proximity (*n* =2)
	Type of physical activity prescribed	Not specified (*n* =3)	Social (*n* =6)	Group Processes
			Environmental (*n* =0)	Group goals (*n*=4)
	Aerobic (*n*=4)			Cooperation (*n*=1)
	Strength Training (*n*=1)		Both (*n* =3)	Competition (*n*=1)
	Not reported (*n*=4)			Interaction and communication (*n* =5)
				Social Support (*n* =5)
				Individual Strategies
				Goal setting (*n* =7)
				Action planning (*n* =2)
				Problem solving (*n* =4)
				Feedback (*n* =3)
				Self-monitoring (*n* =3)
No increase in physical activity (*n*=4)	Intervention Duration	Delivery Staff	Social Cognitive Theory (*n* =2)	Group Structure
	55.50 weeks (±39.75 weeks)	Health professional (*n*=1)	Transtheoretical Model (*n* =2)	Status of group members (*n* =1)
				Group roles (*n* =1)
	Average Total Contact Duration	Trained peer leader (*n*=1)	Social Ecological Theory (*n*=1)	Group norms (*n* =2)
	19.50 hours (±16.97)	Not reported (*n*=2)	Single theory (*n*=1)	Group Environment
				Distinctiveness/Team Identity (*n*=3)
	Fidelity to Classes	Contact Type	Multiple theory (*n*=2)	Group Size (*n* =1)
	26.50 % (±37.47)	In-Person (*n*=4)	Atheoretical (*n*=1)	Leadership (*n* =3)
	Not reported (*n*=2)		Targeted Structural Modification	Proximity (*n* =1)
	Fidelity to Behavioral		Social (*n*=2)	Group Processes
	Components		Environmental (*n*=1)	Interaction and communication (*n* =3)
	62.50 % (±10.67)		Both (*n*=1)	Social Support (*n* =2)
	Not reported (*n*=2)			Individual Strategies
	Type of physical activity prescribed			Goal setting (*n* =2)
	Aerobic and strength training (*n*=1)			Problem solving (*n* =1)
	Not reported (*n*=3)			Self-monitoring (*n* =2)

### Step 5: Data synthesis

**Fig. 3:**
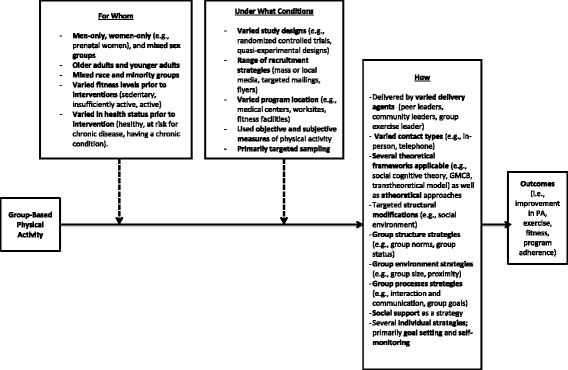
Synopsis of characteristics of effective group-based physical activity promotion interventions

Using the 34 indicators, descriptive summaries were derived based on three categories of studies. These included: (1) Group-based interventions that resulted in a positive effect on participants’ physical activity behavior when compared to individuals or an aggregate of individuals (i.e., not a true group; *n* =39); (2) Studies that compared two true group interventions, both of which significantly increased physical activity behavior (e.g., group-based aerobics versus group-based strength training) but increases were not different between conditions (*n =* 9); and (3) Group dynamics-based intervention studies that did not report positive effects on participants’ behavior outcome(s) (*n* = 4). These three categories are described as ‘effect categories’ for the remainder of the manuscript. A synopsis of the characteristics of the 39 effective interventions is displayed in Fig. 3 and more detailed information is presented below.

### For whom are group-based physical activity interventions effective?

Descriptive summaries of the ‘for whom’ indicators are presented in Table 2. Few eligibility criteria were used across all three effect categories. Overall, almost half of the studies (47 %) targeted populations that were inactive, insufficiently active, or a mix of fitness levels. Notably, 41 % of the effective interventions (i.e., the first two effect categories outlined above; *n =* 48) did not report the pre-intervention fitness level of the target audience. Thirty-one percent of the effective interventions were for women only. Three of the four ineffective interventions targeted primarily minority populations while 83 % of the effective interventions had a mixed racial composition.

With regard to the ‘target population’ indicator within the ‘for whom’ category, interventions that had a positive effect on the outcomes of interest when compared to individuals or an aggregate (i.e., those in the first effect category) described an average of 2.18 (*SD* ±1.57) characteristics of the target sample (e.g., chronic disease, race, sex). Group-based physical activity interventions were found to be effective for older adults (*n =* 10) and, more specifically, older adults at risk for or living with chronic disease (*n =* 4) and older women in a minority group who were also at risk for chronic disease (*n =* 1). A group-based approach was also found to be effective for university students (*n =* 6) as well as a subcategory of women attending university who were at risk for or living with chronic disease (*n =* 2). Other target populations that were positively impacted through participation in a group-based physical activity intervention were individuals with obesity (*n =* 3), with one study delimited to women who were obese. Three studies targeted individuals in minority groups (*n =* 3), with one specifically for women in minority groups. A group-based physical activity intervention (when compared to individuals or an aggregate of individuals) was also effective for adults with chronic conditions (*n =* 1), individuals in low-income categories (n = 1), firepersons (*n =* 1), cancer survivors (*n =* 1), and women in the postnatal period (*n =* 1).

The second effect category (comparison of two true groups) described 2.22 (*SD* ±1.71) characteristics of the sample on average, and targeted individuals at risk for or living with chronic conditions (*n =* 6); specifically for older adults (*n* = 2), adults (*n =* 3), and minorities (*n* = 1). Three interventions also targeted individuals in minority groups, within one specific to women in minority groups.

The interventions that did not have a positive effect on the outcomes of interest (i.e., the final effect category) described an average of 3.75 (*SD* ±2.06) characteristics of the sample. These ‘ineffective’ interventions targeted older adults at risk for or living with chronic disease (*n =* 1), as well as minority groups (*n* = 3). Within the three interventions that targeted minority groups, one study targeted individuals who were also obese and one intervention targeted women who lived in low-income households.

### Under what conditions are group-based physical activity interventions effective?

Fifteen of the group-versus-individual/aggregate interventions (38 %) used pre-post designs and 13 (33 %) were randomized controlled trials. The interventions that compared one true group to another utilized more quasi-experimental designs (44 %) or randomized controlled trials (33 %) than pre-post (11 %) or quasi-experimental without control (11 %) designs. Most of the ineffective interventions were randomized control trials (75 %).

A majority of the studies (75 %) recruited participants through targeted sampling methods. Overall, researchers employed 2.23 (*SD* ±1.41) recruitment strategies; with 2.13 (*SD* ±1.40), 2.44 (*SD* ±1.74), and 2.75 (*SD* ±0.5) recruitment strategies for effective group-versus individual/aggregate interventions, effective true group-versus-true group interventions, and ineffective interventions, respectively. Overall, a low proportion (28 %) of interventions used physician referral, regardless of the target population’s health status (i.e., healthy, at risk, or living with chronic disease). Of those studies that conducted moderation analyses, all five were group-versus-individual/aggregate interventions. Moderation variables are listed in Table 3.

Nine studies (17 %) did not report the specific location of the program. Of the two effective intervention categories (*n =* 48), physical activity programs were effective in the general community, worksites, and fitness facilities. Interestingly, all of the locations for the ineffective interventions were also targeted locations for the interventions that had a positive effect on physical activity behavior outcomes. All four of the ineffective interventions were conducted through in-person delivery. Fifty-four percent of the group-versus-individual/aggregate interventions were delivered through an in-person modality, 28 % were delivered in-person combined with another mode of delivery (e.g., email, telephone), 12.8 % were delivered via telephone, Internet, and/or newsletters, and one study did not report the delivery mode. With regard to the true group-versus-true group comparison category, 44 % of the interventions were delivered in-person, 22 % were delivered in-person plus another mode of delivery, and 34 % did not specify the mode of delivery. The majority of the studies were conducted in the United States; 85 %, 77 %, and 75 % for the group-versus-individual/aggregate category, effective true group-versus-true group category, and ineffective category, respectively. The delivery agent(s) for the effective interventions were often trained peer leaders, community leaders, or group exercise leaders (compared to health professionals or trained research assistants). Delivery agents for the ineffective interventions were health professionals (*n =* 1), trained peer leaders (*n =* 1), or not reported (*n =* 2). Across all three categories, only 13 % (*n* = 7) of the studies reported costs. Of those seven articles, five were effective interventions (71 %). Cost data were frequently vague when mentioned (i.e., authors noted a ‘low cost intervention’ without actual quantitative information related to costs).

Thirty-three percent of all studies reported using both objective and subjective measures (e.g., self-report physical activity and pedometers). Fifty-eight percent of the effective interventions used only a subjective measure of the targeted behavioral outcomes, whereas 100 % of the ineffective interventions used a subjective outcome measure only. A majority (78 %) of effective true group-versus-true group studies, 26 % of the effective group-versus-individual/aggregate interventions, and none of the ineffective interventions used both objective and subjective outcome measures. Few articles included intent-to-treat analyses (23 % across all effect categories).

### How are group-based physical activity interventions effective?

As seen in Table 4, 54 % of all successful interventions did not report a theoretical approach. Eleven (28 %) and seven (18 %) of the successful studies reported using a single or multiple theory approach, respectively. With regard to the successful interventions, 33 % were based on principles of social cognitive theory [1] with others based on the group-mediated cognitive behavioral approach (15 % [5]), social support model (13 %; e.g., principles from Barnes [2]), goal-setting theory (7 %; [42]), team-building model (5 %; [11]), transtheoretical model (5 %; [47]) and social ecological theory (5 %; [6]) and four additional guiding theoretical frameworks were cited once.

Of the nine true group-versus-true group comparison interventions, four were atheoretical, two were developed on the basis of two theories and three used a single guiding theory. Of those interventions that were guided by a theoretical framework, three were based on principles of social cognitive theory (33 %), one used both goal setting and self-determination theory [17], one used the stages of change/transtheoretical model, and one reported the use of a team-building model [11]. Of the four studies that indicated no effect on participants’ targeted behavioral outcomes, one was atheoretical, two used multiple theories, and the fourth intervention used social cognitive theory.

The group-versus-individual/aggregate interventions were shorter in duration than those that compared two true group-based interventions or those that were not effective (see Table 4 for details). Notably, a longer duration did not necessarily indicate a more intensive program over that time. The type of physical activity prescribed to intervention participants, regardless of effect category, was more often aerobic conditioning (35 %) than strength training (3.8 %), or a combination of one or more types of physical activity (7.7 %). Fifty-four percent of the studies did not report the prescribed physical activity type.

Across all studies, an average of 6.68 (*SD* ±2.70) individual and/or group-based strategies were employed in the group-based physical activity interventions (see Table 4 for the full list of strategies). Interestingly, a majority of the effective true group-versus-true group interventions (78 %) had more individualized strategies than group-based strategies. On the other hand, a majority of the group-versus-individual/aggregate interventions used only group-based strategies (74 %) and rarely had more individualized strategies than group-based ones (15 %). The ineffective interventions were more likely to report the use of a greater number of group-based strategies than individual strategies. The most common strategies employed across each effect category can be found in Table 4.

Four of the effective group versus individual/aggregate interventions (12.7 %) used a mediation analysis. Briefly, White et al. [56] found that parental satisfaction mediated the effects of a family-based Internet intervention in relation to measures of body composition (derived from dual-energy X-ray absorptiometry). More recently, authors of two studies conducted mediation analyses for an intervention for women of color: one found that group cohesion mediated the effects of a physical activity program for women of color in relation to increased attendance rates [52] and the other found that task cohesion mediated the effects of a group-based physical activity intervention, designed to support women of color, in relation to improvements in various psychosocial variables (e.g., social support, motivational readiness; [39]). In two studies, Cramp and Brawley [14, 15] found that self-regulatory skills partially mediated the effects of a GMCB intervention, for women in the post-natal period, in relation to post-intervention home-based physical activity. Elliot et al. [20] found that ‘healthy physical activity behaviors’ mediated the relationships between ‘positive physical activity social support’ and ‘physical activity beliefs and understanding’ in relation to measures of overall well-being among a sample of firefighters in the PHLAME study.

### Step 6: Disseminate, implement, and evaluate

The purpose of this realist review was to explore for whom, under what conditions, and how group-based physical activity interventions are effective with regard to physical activity, fitness, exercise, and adherence. All but four of the studies included in this review reported positive effects on these outcome behaviors, corroborating previous findings as to the consistent positive effect of group-based physical activity programs (e.g., [9, 18, 25]). However, the lack of reported ineffective studies also made it difficult to draw many generalizable conclusions *comparing* effective and ineffective interventions. Based on the available data we have surmised observations and recommendations as they relate to the three overarching categories of ‘for whom’, ‘under what conditions’, and ‘how’. While a realist (or evaluative research approach) is difficult to accomplish, the knowledge acquired aligns with real-world application and areas for future research to enhance our understanding of group-based interventions [50].

### Observation 1

Group-based physical activity programs have been effective for a variety of populations; notably for those living with or at risk for chronic disease. This indicates that group-based interventions may be an effective way to increase targeted behavioral outcomes for high-risk populations and those most in need of health behavior intervention. The group-versus-individual/aggregate interventions had more inclusion criteria than exclusion criteria, while the opposite was true of the effective true group-versus-true group interventions or the ineffective interventions. This means that the effective group-based interventions in this review were pragmatic in their eligibility criteria [55], which may increase the potential reach of the intervention. While some populations (i.e., cardiac rehabilitation patients, postnatal women, and individuals who were overweight/obese) were targeted within this literature, there were few studies (<4) conducted within each. In addition, almost half of the interventions did not include the participation rate of those potentially eligible and those who accepted the invitation to join the program. Notably, only 20 % of the interventions used physician referral, while over 50 % of the studies aimed to recruit those at risk for, or living with, a chronic condition.

### Recommendation 1

Future research is needed to explore the boundary conditions related to effective group-based physical activity programs. For example, one study that targeted individuals with prostate cancer was ineffective; however, numerous interventions that targeted those with other chronic conditions were effective. As well, some interventions that targeted minority populations were ineffective, while others were effective. Further exploration of target population subcategories is needed to determine how a group-based program may enhance physical activity behaviors. With regard to interventions that target those who are at-risk for or living with chronic disease, it may be useful to include some level of physician referral. Patients often view their physician as having high credibility related to health behaviors [40]. Finally, this review did not include interventions that targeted youth—a potentially viable research area for group-based physical activity promotion.

### Observation 2

With regard to the conditions that led to the effect of group-based physical activity programs, there was a wide range in intervention durations (i.e., 1 session to 68 weeks with an average of 18 weeks across all interventions). However, about one third of the studies did not report the duration of the intervention. Moreover, there is sparse reporting on costs associated with intervention delivery. Regardless of theoretical basis, the type of physical activity prescribed or the delivery staff, group-based programs were regularly effective. While many of the interventions used trained research assistants to deliver program content, others utilized more sustainable delivery agents such as community leaders, cooperative extension agents, and group exercise leaders. Interestingly, the authors of many of the studies did not report on the frequency by which strategies were delivered to participants. Those that did often reported that sessions containing group-based strategies were held on a weekly basis. However, there were also several studies that indicated that participants met more than once a week and others that reported less frequent meetings. These interventions worked within a variety of settings (e.g., general community, workplaces), and approximately one third of the interventions were conducted at only one site. Again, the data presented here support varied group-based approaches and intensities.

Each of the six studies that tested mediation effects examined different mediators and different outcome variables. In order to further the field of group dynamics as it relates to physical activity promotion, it would be helpful for researchers to test similar mediational pathways across different interventions (i.e., conceptual replication). In fact, each of the six studies operationalized different psychosocial variables and examined their ability to explain the relationship with the outcome of interest (e.g., physical activity engagement, attendance). This variability limits the generalizability of the findings from these unique pathways.

### Recommendation 2

Making use of comparative effectiveness trials may bolster group-based behavioral interventions, as few currently exist in the literature. For example, a factorial design or fractional factorial designs that allow for intervention optimization would provide an opportunity for researchers to compare intervention groups that include multiple, and different combinations of group-based strategies and allows for identification of mediational relationships [13]. For example, there could be conditions *within* both study arms in which some groups receive group feedback and others do not, some complete group goal setting and others do not, and some facilitate a sense of distinctiveness and others do not. In this way, we can isolate the impact of each group-based strategy. It might also be useful to develop hybrid intervention designs, that include both standard ‘randomization’ as well as ‘preference’ arms, in order to determine who might be more attracted to group-based or individually tailored interventions. That is, in a comparative randomized controlled trial, individuals in a ‘preference’ arm (i.e., those who have self-selected into group versus individualized exercise sessions) would be compared to participants who are randomized to either condition (i.e., group- or individually-based exercise groups). Further, information on the degree to which the individual behavior change is initiated and maintained as well as the cost (e.g., time, technology, space) to implement the program should be explored. Other studies may explore more nuanced details on the minimal duration, contact, and strategies used to achieve the effects found in the present review. Minimal interventions have been conducted based on theoretical approaches such as stages of change [46] and social-cognitive theory [19, 53], but less is known about the minimal number of in-person contacts needed for a group-based intervention. That is, some of the group-based interventions were delivered online or via the telephone, with some groups not requiring in-person interaction with other group members [22], and the interventions ranged from one session to over a year of group-based sessions. Understanding how to translate a sense of belonging from an in-person context to other mediums (e.g., website or text-message interventions) may be beneficial as well, especially from cost and sustainability perspectives.

### Observation 3

With regard to how group-based physical activity programs affect behavior, it is evident that the ‘group’ is an effective mode of intervention for fostering physical activity, regardless of study design, recruitment procedures, or analytic procedure employed. In fact, more (54 %) of the effective group-versus-individual/aggregate interventions were atheoretical, but some studies (28 %) used one theory and other studies (18 %) used multiple theories. For the effective group-versus-individual/aggregate interventions, the most commonly employed group-based strategy was interaction through communication. Indeed, both social- and task-based interactions have been shown to influence perceptions of group cohesion [21]. However, less is known about important intervention components such as the most effective combination of strategies to employ with particular populations and in different settings.

### Recommendation 3

We urge group-based physical activity researchers to be transparent in reporting where and when the intervention takes place as well as who delivers it (the latter is reported under ‘how’). Generally speaking, information on the program location or delivery agent was often vague at best (e.g., ‘site-based’, ‘interventionist’). In order to increase the reporting on external validity factors, we support the use of evaluation guidelines such as the Pragmatic-Explanatory Continuum Index Summary (PRECIS; [55]) and the RE-AIM Framework ([31]; RE-AIM.org). When authors use these frameworks, in addition to reporting guidelines (e.g., Consolidated Standards of Reporting Trials [CONSORT]; [44]) researchers and practitioners can more readily share pertinent information related to intervention design and delivery. The disconnect between efficacy trials and their real-world application remains a barrier to health promotion [16, 30]. Transparent and concise reporting of intervention components may speed the rate of translation into sustained practice. Parenthetically, as there were few ineffective interventions (and sparse reporting on setting-level variables in those articles), it was difficult to make comparative recommendations related to setting and staff variables.

### Observation 4

One of the a priori purposes of this study was to provide information on ideal and robust strategies, settings, and delivering agents for effective group dynamics-based intervention by using a realist approach. There were two challenges that thwarted these specific efforts. First, in this study there were too few interventions that did not improve physical activity outcomes, which makes it difficult to isolate what program designers should avoid when developing and delivering these types of interventions. This is especially true as (1) authors often underreported some of the pertinent variables (see Tables 1–3) and (2) variables that were reported in the ineffective studies (e.g., mean age, theoretical underpinnings, strategies employed, and racial composition) were also represented in the effective interventions. For example, related to the lack of reporting, 19 of the 39 effective interventions did not report health status, and of those that did, 12 studies were delivered to those who identified as healthy prior to program participation. There were four ineffective studies two of which included data on the health status of the participants (i.e., ‘those at risk for and with chronic conditions’) and the two remaining ineffective studies did not report health status of participants who attended the group-based interventions. This lack of reporting leaves a gap in our understanding of the boundary conditions for whom these interventions do and do not work. Secondly, while a realist review allows for inference to handle the heterogeneity of research design and outcome measures, we found it challenging to provide any further inferences than our three observations and recommendations as there was heterogeneity (and sometimes disparate groupings) of *all* the factors of interest. This indicates that there is no set formula for group dynamics-based interventions, although participation in these interventions still (largely) improves physical activity behaviors.

### Recommendation 4

Future intervention research remains needed to evaluate the combined effects of individual and contextual factors that may influence physical activity engagement [25].

## Discussion

This study provides information by which ‘the group’ works to improve health behaviors, providing further evidence in support of the notion that participation in a group dynamics-based physical activity program may improve physical activity behaviors. If members of a physical activity group interact, identify as a unit, and express a degree of cohesiveness towards accomplishing goals, they are more likely to succeed [28]. The 48 successful interventions included in this realist review indicate that the positive effect of group-based physical activity programs is pervasive across populations and settings. This is a compelling finding that contradicts typical realist reviews in which researchers typically find that certain strategies work best for particular populations in specified locations [4]. In this realist review we cannot, as we had anticipated at the outset of this study, provide distinctions of what works (and what does not) in the group-based physical activity promotion literature. However, we were able to identify that ‘the group’—as a particular mode of intervention—is effective for most populations and in most settings. That is, the group is a positive mode of intervention to increase physical activity behaviors regardless of whether researchers have targeted different sexes, those with disparate health statuses, used one or multiple group-based strategies, or conducted the intervention in a workplace or in the community at large.

One limitation of the present study relates to the quality appraisal score. In response to Pawson et al.’s [45] critique of current quality appraisal metrics, we developed two dichotomous ratings: one for ‘rigor’ and one for ‘relevance’. These two items were used to determine if the evidence we found was relevant to the study of group-based strategies to increase physical activity behaviors and if the authors’ conclusions aligned with their research question and design. We acknowledge that these two items have not undergone previous evaluation and testing. That said, these items fit the realist paradigm and allow for a broader use of the literature (e.g., qualitative studies, case studies). Another limitation of the present study is that realist reviews do not account for sample size and effect sizes. However, the results complement the findings of the previous meta-analysis of group-based physical activity interventions [9]. It has been almost a decade since the publication of Burke and colleagues’ meta-analysis, which may warrant an updated data synthesis that includes more recent group dynamics-based physical activity intervention studies. Finally, the proportion of successful interventions is not entirely surprising as journals often publish interventions that found significant effects on physical activity and exclude those that had null findings (i.e., publication bias). While difficult to obtain, the inclusion of data regarding what has not worked in efficacy or effectiveness trials would significantly contribute to this body of evidence.

## Conclusions

The current study contributes to the body of literature in three ways. First, by using a realist review methodology, we were able to combine theoretical approaches as well as use terms from organizational psychology for an exhaustive search that expanded the inclusion criteria from a previous systematic review of group-based physical activity programs that used Carron and Spink’s [11] team-building model [25]. Second, we propose herein three recommendations for research that relate to each realist dimension (for whom, under what conditions, and how). Third, this realist review highlights gaps around appropriate group-based strategies for particular contexts and populations, and provides potential avenues for related future research. By addressing these gaps, researchers and interventionists will be able to more fully understand the influence of the group on physical activity behavior change.

## Availability of data and materials

Not applicable.

## Additional files

Additional file 1:
**Eligibility Criteria.** Inclusion criteria for articles. A tabular representation of the criteria for inclusion in the realist review. (PDF 71 kb) Additional file 2:
**References.** Supplemental Reference List: Articles included in realist review, not referenced in text. All articles included in realist review, if not referenced within the text. (PDF 100 kb)

## Abbreviations

GBPA: Group-based physical activity
GMCB: Group-mediated cognitive behavioral
