# Supplementary material for: Understanding for whom, under what conditions, and how group-based physical activity interventions are successful: a realist review
Source: BMC Public Health. 2015 Sep 24;15:958. doi: 10.1186/s12889-015-2270-8 (PMC4582831; doi:10.1186/s12889-015-2270-8)
Supplement: Additional file 1: — Eligibility Criteria. Inclusion criteria for articles. A tabular representation of the criteria for inclusion in the realist review. (PDF 71 kb) [file 12889_2015_2270_MOESM1_ESM.pdf]

Supplemental Table
Inclusion criteria for articles

**Data type**	**Inclusion criteria**
Participants	- >17 years of age
Language	- Available in English language
Study design	- Used experimental or quasi-experimental design<br>- Qualitative analysis of a physical activity intervention
Control condition	- Any comparator including active control, inactive control, or pre and post-measure
Intervention	- Explicit statement of the use of at least one group-based strategy
Primary outcome(s) (at least one of these outcomes)	- Physical activity<br>- Exercise<br>- Fitness<br>- Adherence<br>- Qualitative measure of physical activity behaviors
