# Supplementary material for: Understanding for whom, under what conditions, and how group-based physical activity interventions are successful: a realist review
Source: BMC Public Health. 2015 Sep 24;15:958. doi: 10.1186/s12889-015-2270-8 (PMC4582831; doi:10.1186/s12889-015-2270-8)
Supplement: Additional file 2: — References. Supplemental Reference List: Articles included in realist review, not referenced in text. All articles included in realist review, if not referenced within the text. (PDF 100 kb) [file 12889_2015_2270_MOESM2_ESM.pdf]

Supplemental Reference List: Articles included in realist review, not referenced in text

Ard, JD, Kumanyika, S, Stevens, VJ, Vollmer, WM, Samuel-Hodge, C, Kennedy, B, . . . Svetkey, LP. (2008). Effect of group racial composition on weight loss in African Americans. *Obesity* (*Silver Spring), 16*(2), 306-310. doi: 10.1038/oby.2007.49

Barrera, M, Strycker, LA, Mackinnon, DP & Toobert, DJ. (2008). Social-ecological resources as mediators of two-year diet and physical activity outcomes in type 2 diabetes patients. *Health Psychology, 27*(2 Suppl), S118-125. doi: 10.1037/0278-6133.27.2(Suppl.).S118

Befort, CA, Klemp, JR, Austin, HL, Perri, MG, Schmitz, KH, Sullivan, DK, & Fabian, CJ. (2012). Outcomes of a weight loss intervention among rural breast cancer survivors. *Breast Cancer Research and Treatment*, *132*(2), 631-639. doi: 10.1007/s10549-011-1922-3

Blake, SM, Caspersen, CJ, Finnegan, J, Crow, RA, Mittlemark, MB, & Ringhofer, KR. (1996). The shape up challenge: a community-based worksite exercise competition*.* *American Journal of Health Promotion, 11*(1), 23-34.

Bopp, M, Wilcox, S, Laken, M, Hooker, SP, Parra-Medina, D, Saunders, R, . . . McClorin, L. (2009). 8 Steps to Fitness: a faith-based, behavior change physical activity intervention for African Americans*. Journal of Physical Activity and Health, 6*(5), 568-577.

Brawley, LR, Arbour-Nicitopoulos, KP, & Martin Ginis, KA (2013). Developing physical activity interventions for adults with spinal cord injury. Part 3: a pilot feasibility study of an intervention to increase self-managed physical activity. *Rehabilitation Psychology*, *58*(3), 316-321. doi: 10.1037/a0032814

Buis, LR, Poulton, TA, Holleman, RG, Sen, A, Resnick, PJ, Goodrich, DE . . . Richardson, CR. (2009). Evaluating Active U: an Internet-mediated physical activity program. *BMC Public Health, 9*, 331. doi: 10.1186/1471-2458-9-331

Buman, MP, Giacobbi, PR, Jr, Dzierzewski, JM, Aiken Morgan, A, McCrae, CS, Roberts, BL, & Marsiske, M. (2011). Peer volunteers improve long-term maintenance of physical activity with older adults: a randomized controlled trial. *Journal of Physical Activity and Health*, *8* Suppl 2, S257-266.

Carmack Taylor, CL, Demoor, C, Smith, MA Dunn, AL, Basen-Engquist, K, Nielsen, I, . . . Gritz, ER. (2006). Active for Life After Cancer: a randomized trial examining a lifestyle physical activity program for prostate cancer patients. *Psychooncology*, *15*(10), 847-862. doi: 10.1002/pon.1023

Carter, CL, Onicescu, G, Cartmell, KB, Sterba, KR, Tomsic, J, & Alberg, AJ. (2012). The comparative effectiveness of a team-based versus group-based physical activity intervention for cancer survivors. *Support Care Cancer, 20*(8), 1699-1707. doi: 10.1007/s00520-011-1263-0

Courneya, KS, Blanchard, CM, & Laing, DM. (2001). Exercise adherence in breast cancer survivors training for a dragon boat race competition: a preliminary investigation. *Psychooncology, 10*(5), 444-452.

Cramp, AG, & Brawley, LR. (2006). Moms in motion: a group-mediated cognitive-behavioral physical activity intervention. *International Journal of Behavioral Nutrition and Physical Activity, 3*, 23. doi: 10.1186/1479-5868-3-23

Cramp, A.G, & Brawley, LR. (2009). Sustaining self-regulatory efficacy and psychological outcome expectations for postnatal exercise: effects of a group-mediated cognitive behavioural intervention. *British Journal of Health Psychology, 14*(Pt 3), 595-611. doi: 10.1348/135910708x383732

Elliot, DL, Goldberg, L, Kuehl, KS, Moe, EL, Breger, RK, & Pickering, MA. (2007). The PHLAME (Promoting Healthy Lifestyles: Alternative Models' Effects) firefighter study: outcomes of two models of behavior change. *Journal of Occupational and Environmental Medicine, 49*(2), 204-213. doi: 10.1097/JOM.0b013e3180329a8d

Estabrooks, PA, Bradshaw, M, Dzewaltowski, DA, & Smith-Ray, RL (2008). Determining the impact of Walk Kansas: applying a team-building approach to community physical activity promotion. *Annals of Behavioral Medicine, 36*(1), 1-12. doi: 10.1007/s12160-008-9040-0

Fisher, K. J., & Li, F. (2004). A community-based walking trial to improve neighborhood quality of life in older adults: a multilevel analysis. *Annals of Behavioral Medicine*, 28(3), 186-194. doi: 10.1207/s15324796abm2803_7

Focht, BC, Garver, MJ Devor, S.T, Dials, J. Rose, M, Lucas, AR, . . . Rejeski, WJ. (2012). Improving maintenance of physical activity in older, knee osteoarthritis patients trial-pilot (IMPACT-P): design and methods. *Contemporary Clinical Trials, 33*(5), 976-982. doi: 10.1016/j.cct.2012.04.012

Foy, CG, Wickley, KL, Adair, N, Lang, W, Miller, ME, Rejeski, WJ, . . . Berry, MJ. (2006). The Reconditioning Exercise and Chronic Obstructive Pulmonary Disease Trial II (REACT II): rationale and study design for a clinical trial of physical activity among individuals with chronic obstructive pulmonary disease. *Contemporary Clinical Trials, 27*(2), 135-146. doi: 10.1016/j.cct.2005.11.011

French, SA, Harnack, LJ, Hannan, PJ, Mitchell, NR, Gerlach, AF & Toomey, TL. (2010). Worksite environment intervention to prevent obesity among metropolitan transit workers. *Preventive Medicine, 50*(4), 180-185. doi: 10.1016/j.ypmed.2010.01.002

Gaston, MH, Porter, GK, & Thomas, VG. (2007). Prime Time Sister Circles: evaluating a gender-specific, culturally relevant health intervention to decrease major risk factors in mid-life African-American women. *Journal of the National Medical Association, 99*(4), 428-438.

Gokee LaRose, J, Leahey, TM, Weinberg, BM, Kumar, R, & Wing, RR. (2012). Young adults' performance in a low-intensity weight loss campaign. *Obesity (Silver Spring), 20*(11), 2314-2316. doi: 10.1038/oby.2012.30

Green, BB, Cheadle, A, Pellegrini, AS, & Harris, JR. (2007). Active for life: a work-based physical activity program. *Preventing Chronic Disease, 4*(3), A63.

Hooker, SP, Harmon, B, Burroughs, EL, Rheaume, CE, & Wilcox, S. (2011). Exploring the feasibility of a physical activity intervention for midlife African American men. *Health Education Research, 26*(4), 732-738. doi: 10.1093/her/cyr034

Hughes, SL, Seymour, RB, Campbell, R, Pollak, N, Huber, G, & Sharma, L. (2004). Impact of the fit and strong intervention on older adults with osteoarthritis. *Gerontologist, 44*(2), 217-228.

Ince, ML. (2008). Use of a social cognitive theory-based physical-activity intervention on health-promoting behaviors of university students. *Perceptual and Motor Skills, 107*(3), 833-836. doi: 10.2466/pms.107.3.833-836

Izquierdo-Porrera, AM, Powell, CC, Reiner, J, & Fontaine, KR. (2002). Correlates of exercise adherence in an African American church community. *Cultural Diversity and Ethnic Minor Psychology, 8*(4), 389-394.

Jancey, JM, Lee, AH, Howat, PA, Clarke, A, Wang, K, & Shilton, T. (2008). The effectiveness of a physical activity intervention for seniors. *American Journal of Health Promotion, 22*(5), 318-321. doi: 10.4278/ajhp.22.5.318

Katula, JA, Sipe, M, Rejeski, WJ, & Focht, BC. (2006). Strength training in older adults: an empowering intervention. *Medicine and Science in Sports and Exercise, 38*(1), 106-111.

Kim, KH, Linnan, L, Campbell, MK, Brooks, C, Koenig, HG, & Wiesen, C. (2008). The WORD (wholeness, oneness, righteousness, deliverance): a faith-based weight-loss program utilizing a community-based participatory research approach. *Health Education and Behavior, 35*(5), 634-650. doi: 10.1177/1090198106291985

King, AC, Carl, F, Birkel, L, & Haskell, WL. (1988). Increasing exercise among blue-collar employees: the tailoring of worksite programs to meet specific needs. *Preventive Medicine, 17*(3), 357-365.

Klug, C, Toobert, DJ, & Fogerty, M. (2008). Healthy Changes for living with diabetes: an evidence-based community diabetes self-management program. *Diabetes Education, 34*(6), 1053-1061. doi: 10.1177/0145721708325886

Leahey, TM, Crane, MM, Pinto, AM, Weinberg, B, Kumar, R, & Wing, RR. (2010). Effect of teammates on changes in physical activity in a statewide campaign. *Preventive Medicine, 51*(1), 45-49. doi: 10.1016/j.ypmed.2010.04.004

Lee, RE, O'Connor, DP, Smith-Ray, R, Mama, SK, Medina, AV, Reese-Smith, JY . . . Estabrooks, PA. (2012). Mediating effects of group cohesion on physical activity and diet in women of color: health is power. *American Journal of Health Promotion, 26*(4), e116-125. doi: 10.4278/ajhp.101215-QUAN-400

Leermakers, EA, Perri, MG, Shigaki, CL, & Fuller, PR. (1999). Effects of exercise-focused versus weight-focused maintenance programs on the management of obesity. *Addictive Behaviors, 24*(2), 219-227.

Lombard, C, Deeks, A, Jolley, D, Ball, K, & Teede, H. (2010). A low intensity, community based lifestyle programme to prevent weight gain in women with young children: cluster randomised controlled trial*. BMJ, 341*, c3215. doi: 10.1136/bmj.c3215

Marinescu, LG, Sharify, D, Krieger, J, Saelens, BE, Calleja, J, & Aden, A. (2013). Be active together: supporting physical activity in public housing communities through women-only programs. *Progress in Community Health Partnerships, 7*(1), 57-66. doi: 10.1353/cpr.2013.0003

Moore, S. M., & Charvat, J. M. (2002). Using the CHANGE intervention to enhance long-term exercise. *Nursing Clinics of North America, 37*(2), 273-283, vi-vii.

Morris, A, Do, D, Gottlieb-Smith, R, Ng, J, Jain, A, Wright, S, & Shochet, R. (2012). Impact of a fitness intervention on medical students. *Southern Medical Journal, 105*(12), 630-634. doi: 10.1097/SMJ.0b013e318273a766

Netz, Y, Axelrad, S, & Argov, E. (2007). Group physical activity for demented older adults feasibility and effectiveness. *Clinical Rehabilitation, 21*(11), 977-986. doi: 10.1177/0269215507078318

Perri, MG, Martin, AD, Leermakers, EA, Sears, SF, & Notelovitz, M. (1997). Effects of group- versus home-based exercise in the treatment of obesity. *Journal of Consulting and Clinical Psychology, 65*(2), 278-285.

Peterson, JA, Yates, BC, Atwood, JR, & Hertzog, M. (2005). Effects of a physical activity intervention for women. *Western Journal of Nursing Research, 27*(1), 93-110. doi: 10.1177/0193945904270912

Rejeski, WJ, Brawley, LR, Ambrosius, WT, Brubaker, PH, Focht, BC, Foy, CG, & Fox, LD. (2003). Older adults with chronic disease: benefits of group-mediated counseling in the promotion of physically active lifestyles. *Health Psychology, 22*(4), 414-423.

Rejeski, WJ, Mihalko, SL, Ambrosius, WT, Bearon, LB, & McClelland, JW. (2011). Weight loss and self-regulatory eating efficacy in older adults: the cooperative lifestyle intervention program. *Journals of Gerontology, Series B: Psychological Sciences and Social Sciences, 66*(3), 279-286. doi: 10.1093/geronb/gbq104

Rhodes, RE, Martin, AD, & Taunton, JE. (2001). Temporal relationships of self-efficacy and social support as predictors of adherence in a 6-month strength-training program for older women. *Perceptual and Motor Skills, 93*(3), 693-703. doi: 10.2466/pms.2001.93.3.693

Rimmer, JH, Rauworth, A, Wang, E, Heckerling, PS, & Gerber, BS. (2009). A randomized controlled trial to increase physical activity and reduce obesity in a predominantly African American group of women with mobility disabilities and severe obesity. *Preventive Medicine, 48*(5), 473-479.

Smith-Ray, RL, Mama, S, Reese-Smith, JY, Estabrooks, PA, & Lee, RE. (2012). Improving participation rates for women of color in health research: the role of group cohesion. *Prevention Science, 13*(1), 27-35. doi: 10.1007/s11121-011-0241-6

Stoffelmayr, BE, Mavis, BE, Stachnik, T, Robison, J, Rogers, M, VanHuss, W, & Carlson, J. (1992). A program model to enhance adherence in work-site-based fitness programs. *Journal of Occupational Medicine, 34*(2), 156-161.

Tan, EJ, Rebok, GW, Yu, Q, Frangakis, CE, Carlson, MC, Wang, T, . . . Fried, LP. (2009). The long-term relationship between high-intensity volunteering and physical activity in older African American women. *Journals of Gerontology, Series B: Psychological Sciences and Social Sciences, 64*(2), 304-311. doi: 10.1093/geronb/gbn023

Thompson, JL, Allen, P, Helitzer, DL, Qualls, C, Whyte, AN, Wolfe, VK, & Herman, CJ. (2008). Reducing diabetes risk in American Indian women. *American Journal of Preventive Medicine, 34*(3), 192-201. doi: 10.1016/j.amepre.2007.11.014

Toobert, DJ, Glasgow, RE, Strycker, LA, Barrera, M, Jr, Ritzwoller, DP, & Weidner, G. (2007). Long-term effects of the Mediterranean lifestyle program: a randomized clinical trial for postmenopausal women with type 2 diabetes*. International Journal of Behavioral Nutririon and Physical Activity, 4*, 1. doi: 10.1186/1479-5868-4-1

Toobert, DJ, Strycker, LA, Glasgow, RE, Barrera Jr, M, & Angell, K. (2005). Effects of the mediterranean lifestyle program on multiple risk behaviors and psychosocial

outcomes among women at risk for heart disease. *Annals of Behavioral Medicine, 29*(2), 128-137. doi: 10.1207/s15324796abm2902_7

Wilcox, S, Dowda, M, Leviton, LC, Bartlett-Prescott, J, Bazzarre, T, Campbell-Voytal, K., . . . Wegley, S. (2008). Active for life: final results from the translation of two physical activity programs. *American Journal of Preventive Medicine, 35*(4), 340-351. doi: 10.1016/j.amepre.2008.07.001

Wilson, MG, Basta, TB, Bynum, BH, DeJoy, DM, Vandenberg, RJ, & Dishman, RK. (2010). Do intervention fidelity and dose influence outcomes? Results from the move to improve worksite physical activity program. *Health Education Research, 25*(2), 294-305. doi: 10.1093/her/cyn065

Young, DR, & Stewart, KJ. (2006). A church-based physical activity intervention for African American women. *Family and Community Health, 29*(2), 103-117.
